# Cosmeceutical Potential of Major Tropical and Subtropical Fruit By-Products for a Sustainable Revalorization

**DOI:** 10.3390/antiox11020203

**Published:** 2022-01-21

**Authors:** Abigail García-Villegas, Alejandro Rojas-García, María del Carmen Villegas-Aguilar, Patricia Fernández-Moreno, Álvaro Fernández-Ochoa, María de la Luz Cádiz-Gurrea, David Arráez-Román, Antonio Segura-Carretero

**Affiliations:** 1 Department of Analytical Chemistry, University of Granada, 18071 Granada, Spain; abigarcia@ugr.es (A.G.-V.); alejorogar@ugr.es (A.R.-G.); marivillegas@ugr.es (M.d.C.V.-A.); patrifdez@correo.ugr.es (P.F.-M.); darraez@ugr.es (D.A.-R.); ansegura@ugr.es (A.S.-C.); 2 Max Delbrück Center for Molecular Medicine in the Helmholtz Association, 13125 Berlin, Germany; alvaro.fernandezochoa@mdc-berlin.de; 3 Berlin Institute of Health Metabolomics Platform, 13125 Berlin, Germany

**Keywords:** tropical fruits, by-products, phenolic compounds, green extraction, cosmeceuticals, skin health

## Abstract

The increasing production of tropical fruits followed by their processing results in tons of waste, such as skins or seeds. However, these by-products have been reported to be rich in bioactive compounds (BACs) with excellent properties of interest in the cosmeceutical industry: antioxidant, anti-aging, anti-inflammatory, antimicrobial and photoprotective properties. This review summarizes the tropical fruits most produced worldwide, their bioactive composition and the most important and studied therapeutic properties that their by-products can contribute to skin health, as well as the different approaches for obtaining these compounds using techniques by conventional (Soxhlet, liquid-liquid extraction or maceration) and non-conventional extractions (supercritical fluid extraction (SFE), ultrasound-assisted extraction (UAE), microwave-assisted extraction (MAE), pressurized liquid extraction (PLE) and two-phase aqueous system), followed by their identification by HPLC-MS or GC-MS analysis. Moreover, this work encompasses several studies that may prove the effects of seeds and skins from tropical fruits against oxidative stress, hyperpigmentation, acne, aging or UV radiation. Therefore, the investigation of functional components present in tropical fruit by-products under a circular bioeconomy model could be of great interest for the cosmeceutical industry and a very promising option for obtaining new cosmeceutical formulations.

## 1. Introduction

In recent years, the production of tropical fruits has increased considerably. Although tropical fruits only account for 3% of the global exports of agricultural food products, they are in the third most valuable group of fruits worldwide. Currently, the main tropical fruits marketed are mango, pineapple, avocado and papaya, which occupy the largest volume of production [1]. However, there is also the small production of other lesser-known fruits, such as cherimoya, guava, passion fruit and litchi [2]. Their high production and demand are due to the proven health benefits, in addition to their flavor and exotic character. Besides their contribution to macronutrients consumption, tropical fruits are characterized as a natural source of BACs with high antioxidant capacity associated with the prevention of diseases related to oxidative stress, aging and chronic inflammation, which is why there is great interest in them from the food, pharmaceutical and cosmeceutical industry [3]. Within these BACs, phenolic compounds must be highlighted, especially phenolic acids, flavonoids, flavan-3-ols, anthocyanidins, coumarins and isoflavonoids [4,5,6].

As said before, tropical fruits are highly demanded and produced all around the world, which leads to a problematic waste generation. Estimations reflect that approximately 30% of the world’s food is wasted or lost by the end of the year [7]. In terms of environmental impact, food waste generates 8% of the global greenhouse gas (GHG) emissions [8]. The solution to these problems could be the application of a circular bioeconomy model, through which all this waste is reused and revalorized, creating high value-added products. Currently, many studies are being carried out on the bioactive characteristics of by-products from tropical fruits, such as mango, avocado, papaya and cherimoya, among others [9,10,11,12]. It has been observed that by-products such as skin, seeds and leaves often contain higher amounts of phenolic compounds than edible parts; for instance, up to 15% more in avocado skin and seed than pulp. Thus, they may be interesting as sources of bioactive ingredients in order to apply them in new value-added products [13].

Therefore, tropical fruit by-products can be considered as new and promising choices to obtain BACs. The way they are obtained is crucial: the extraction process is the most important step for the recovery of these compounds. This process can be treated from different approaches: conventional extraction techniques, such as maceration and Soxhlet extraction, and non-conventional techniques, such as UAE, MAE or subcritical water extraction (SWE). The main differences appreciated are price, extraction times, usage of organic solvents and extraction efficiency. In addition, these suitable, inexpensive and environmentally friendly technologies use non-toxic solvents recognized as safe (GRAS) in order to reduce pollution maintaining extraction efficiency, being recognized as the best option to be chosen [14,15,16]. Extraction can promote the growth of cosmetic industries, which are interested in sustainable and cheap sources of functional ingredients. In fact, the main benefits found in these ingredients in the cosmeceutical field are due to the antioxidant, anti-aging, anti-inflammatory, antimicrobial and photoprotective activities of the contained BACs. The mechanisms of action of these compounds in skin care are based on neutralizing free radicals that induce harmful effects, such as edema, erythema, photoaging, hyperpigmentation and skin cancer [2]. Therefore, an interesting strategy for reusing food by-products rich in bioactives is the development of cosmeceuticals with a therapeutic action capable of affecting the skin in a positive way beyond the time of its application [17].

In the present work, a review is carried out about the BACs present in the main tropical fruits and their by-products with the aim of determining their benefits for the skin, taking into account the recovery methods and the main therapeutic targets for the development of cosmeceuticals.

## 2. Material and Methods

All the scientific information has been collected from literature included in the databases Google Scholar, PubMed and Scopus. The process consisted in selecting available studies published during the last 5 years, so the chosen timeline for the main searching was between 2016 and 2021. However, works from other years have been included for their relevance. 

Our main topics selected were “Tropical Fruits”, “Avocado”, “Mango”, “Pineapple”, “Papaya”, “Litchi”, “Guava”, “Custard Apple”, “Passion Fruit” (and all their corresponding Latin names), “By-products”, “Bioactive Compounds”, “Phenolic Compounds”, “Polyphenols”, “Skin Health”, “Skin Aging”, “Photoprotection”, “Skin Cancer”, “Skin Hyperpigmentation”, “Wound Healing”, “Acne”, “Cosmeceuticals” and “Green/Novel Extraction”. Right from the start, using (Tropical Fruits AND Bioactive Compounds AND Skin Health), the fruits investigated in this review highlighted, either because of their high production/consumption, its outstanding antioxidant capacity, its extensive use in cosmetic products over the years, or even all of these combined. On the other hand, the leading therapeutic targets where tropical fruit bioactives are involved, theoretically or experimentally, in the promotion of skin health were also pointed out. Moreover, extraction techniques performed on those papers were revised as well. From that moment on, search terms were set, combining themselves as follows: (avocado or mango or pineapple or papaya or guava or custard apple or passion fruit or litchi) AND (by-products) AND (phenolic compounds) AND (skin aging or photoprotection or skin cancer or hyperpigmentation or wound healing or acne). In most cases, search terms were shortened in order to go more deeply into the subject, specifically choosing only one fruit or one skin therapeutic target, or both. 

Finally, for more detailed analysis, some inclusion and exclusion criteria were taken into account. Articles, reviews and book chapters were all included, and even different scientific magazine publications or international, national or statal reports. Moreover, articles were admitted even if the cosmetic formulation was finally created or only therapeutic activities were measured on the extract itself without the physical preparation of the cosmetic. Concerning communication language, we stablished some restrictions: only English papers were legitimately admitted, allowing those with an abstract in their natal language. Other additional aspects were also treated, e.g., non-conventional extraction techniques, extraction of other types of BACs, such as carotenoids, and different vehicles (encapsulation and liposomes) for a suitable delivery of the bioactive ingredient into the skin.

## 3. Agronomic Characteristics of Main Tropical and Subtropical Fruits

Global tropical and subtropical fruit production has grown considerably over the past decade in response to recent interest from importing countries outside tropical and subtropical areas (mainly European countries). This is mainly due to its attractive sensory characteristics, exotic character and undeniable value as a promoter of health in humans [2]. This is why their importance at the nutritional level (implanted in a large number of diets worldwide) and economic level (they are considered the most valuable fruits after bananas and apples) has increased enormously [1].

**Figure 1:**
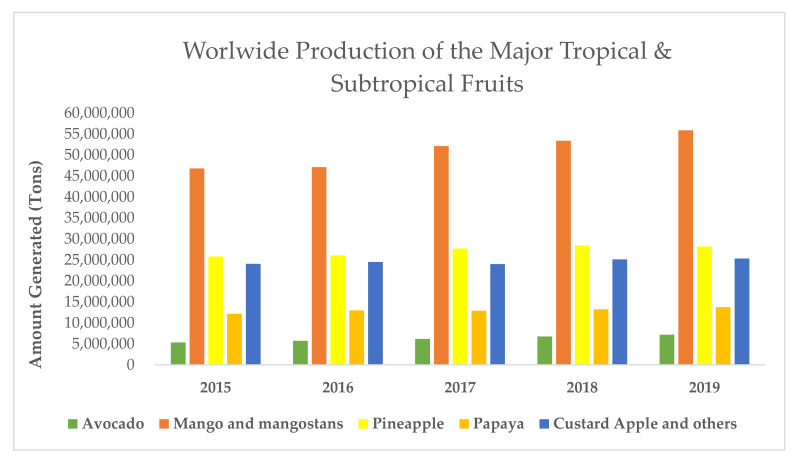
Worldwide production of the major tropical and subtropical fruits.

Observing production data in Figure 1, mangoes, pineapples, papayas and avocados are considered the main tropical fruits, while other fruits, such as lychee, passion fruit or chirimoya, make up the minority [18].

### 3.1. Mango (Mangifera indica L.)

Mango is one of the most recognized tropical fruits and the fifth most produced fruit in the world, as it is grown in more than 90 countries and with more than 38.9 million tons produced per year [19]. Mexico, Indonesia, India, Pakistan, Brazil, the Philippines, Nigeria and Egypt have been the lead countries since the outset, although over the past few decades, its production has developed in other areas, such as the south-eastern US, the rest of South America, Australia, Hawaii, the Mediterranean basin, etc. [20].

The fruit belongs to the family *Anacardiaceae* and its tree, which grows quickly, can exceed 30 m in height and diameter. Its leaves, oblong and lanceolate, acquire a bright dark green color and are between 15 and 40 cm large. Its yellowish or reddish flowers grow in clusters 30 cm long. Its fruit, the mango itself, is a drupe of variable morphology depending on the type, with a smooth, dotted and thin but resistant skin. The coloration of the fruit passes from green to yellow, orange or purple red, partially or homogeneously. In total, the fruit can weigh between 130 g and more than one kilogram, depending on the variety. The pulp has an orange-yellow hue with a lot of aroma and a powerful flavor, and it is very juicy [21].

Inside, right in the center, is a large, flattened, cornered seed, of which the outer side sprouts and prolongs fibers that penetrate the mesocarp and attach it to the pulp. This seed is composed of an endocarp (seed cover) that encloses and protects a species of almond called kernel [22].

In terms of mango production, as it is the market leader in tropical and subtropical fruits, the waste generation associated with it is enormous. These are mainly its seeds (kernels and wrapping), which make up between 20 and 60% of the total weight of the fruit and generate more than 123,000 tons per year, and the skin, which accounts for between 7% and 24% [22]. In total, it is estimated that between 35 and 60% of the total weight of the fruit is discarded in seeds and skins [23], which represents an immense loss at the nutritional level for its richness, and economic for its multiple options of revaluation [20].

In terms of its nutritional composition, depending on the variety of mango, production conditions or maturity, mango pulp is usually the source of the same macronutrients, such as carbohydrates, proteins, amino acids, lipids, fiber and organic acids, as well as considerable richness in micronutrients, such as vitamins A, B and C, minerals, such as calcium, magnesium, potassium, sodium, phosphorus or iron, and BACs, such as carotenoids, phenolic acids (ferulic, chlorogenic and gallic mainly), phyto- and tocosterols, alkaloids and numerous polyphenols (gallotannins, flavonols, benzophenols and their derivatives: maclurin and iriflofenone, mangiferin, homomangiferin, isomangiferin, anthocyanins, kaempferol and quercetin) [24,25].

### 3.2. Avocado (Persea americana L.)

The avocado (*Persea americana*) is an important oleaginous fruit native to Central America, which was first cultivated in Mexico in 500 BC. [26]. It belongs to the *Lauraceae* family and the *Persea* genus and is produced mainly in tropical and subtropical areas with warm and temperate climates, although it is currently cultivated throughout the world. The Hass and Fuerte varieties are the most consumed and those that dominate the international market [27]. The avocado fruit is made up of the epicardium (skin), mesocarp (pulp) and endocarp (seed), of which the shape, size, color and phytochemical content depend on the genotype and to a lesser extent on environmental and geographical conditions. It has approximately 136 g of edible fruit, which is characterized by having a light green color and a smooth and creamy texture covered by a thick, rough and dark green skin. The skin and the seed represent approximately 33% of the total weight of the fruit [28]. Avocado is normally consumed as fresh fruit, but in recent years, industrial products such as guacamole and avocado oil have appeared, which generate a large number of by-products [29].

Due to global demand, avocado production reached 6.3 million tons in 2018, increasing 6.7% compared to 2017. More than half of the total production comes from Central America and the Caribbean, where Mexico is the main avocado producer. Of all the main tropical fruits, avocado is the one that has experienced the greatest growth in production in recent years. In 2018, around 35% of the global avocado production was exported mainly to the European Union and North America [30]. By 2029, avocado production is expected to slightly exceed 11 million tons [1].

The avocado is characterized by being a fruit with a high nutritional quality with health benefits [26]. Avocado pulp is a good source of protein, carbohydrates, dietary fiber, minerals, such as potassium and magnesium, and antioxidant vitamins, such as vitamin C and E, vitamin K1 and vitamins of group B. It is rich in phytochemicals, such as carotenoids (xanthophylls and B-carotenes), that are important in reducing oxidative stress. In addition, it contains phenolic compounds beneficial to health that reduce oxidative stress and have anti-inflammatory activity. However, avocado pulp is recognized for its high content of monounsaturated and polyunsaturated fatty acids that are beneficial for cardiovascular health [27]. 

### 3.3. Pineapple (Ananas comosus L.)

Pineapple, a monocotyledonous perennial plant belonging to the *Bromeliaceae* family, offers a tropical fruit of the same name with a high organoleptic and nutritional appeal thanks to the balance of acidity and sweetness of its flavor. It is cultivated mostly in countries such as the Philippines, Thailand, Indonesia, Malaysia, Kenya, India and China. It is the most processed tropical fruit, which has led to a gradual increase in its production over the decades [31] In addition, its importance in food production and its pharmacological and cosmetic utility makes it the second most popular plant in the world [32].

Its annual production is estimated at about 29.5 million tons per year, mainly intended for the production of salads, essences, juices, jams and canned fruits [33]. The by-products generated from this production account for almost 60% of the total weight of fresh fruit, mainly between 29 and 42% for the skin, and between 9 and 20% for the core [34]. This generation of waste, as in mango and avocado, causes a high impact from a nutritional and economic point of view. Therefore, it includes the growing interest in the treatment and revalorization of these wastes for the obtaining of new products with high added value.

Pineapple is an important source of sugars, organic acids, essential minerals, fibers and vitamins for human nutrition [35], as well as various biomolecules of high commercial interest, with enzymes (especially bromelain) being the most recognized. Bromelain (EC 3.4.22.33) is a proteolytic enzyme belonging to the cysteine group, whose concentration in the stem and skin exceeds that in the pulp. Among its numerous applications, it is worth highlighting its nutraceutical, pharmacological and cosmetic profile thanks to its anti-inflammatory activity, joint support and digestion support by promoting protein rupture [35,36].

### 3.4. Papaya (Carica papaya L.)

*Carica papaya* L. is the most popular and economically most important species of the family *Caricaceae*. The *C. papaya* tree is native to Central and South America and is one of the main fruit plants grown in the world, especially in tropical and subtropical areas. It is a semi-woody herbaceous plant with large, fast-growing palmate leaves that can reach 12 m in height. The production of its fruit occurs throughout the year and each fruit usually weighs between 1 and 3 kg. Papaya is a fruit that is characterized by having an almost elongated oval shape and as it matures, its color ranges from green to intense yellow. It has a thin but quite hard skin, a fleshy, orange and sweet pulp and a central cavity where we find numerous small, black and round seeds [37]. Although papaya is grown and consumed mainly in developing countries, it is becoming an important fruit internationally, both as a fresh fruit and as a processed product.

According to the Food and Agriculture Organization of the United Nations (FAO), the global production of papaya was approximately 13 million tons in 2016, where India and Brazil were the main producers with 5.7 and 1.4 million tons respectively. In 2017, the global papaya production amounted to 13.3 million tons, 2.7% more than in 2016, where despite bad weather conditions, papaya crops suffered considerably less damage than other tropical fruits [38]. Papaya production in 2018 reached a figure of 13.6 million tons, approximately 4% more than in 2017, with India being the largest producer and Mexico the main exporter [30]. Regarding future projections, it is estimated that the global papaya production will increase at a rate of 2.1% per year, reaching 16.6 million tons in 2029 [39]. 

In 2019, the global papaya exports increased by 3.2%, reaching approximately 349,000 tons, reflecting a clear recovery from the decreases produced in 2017 and 2018 due to the weather. Currently, Mexico is the main exporter of papayas in the world, with approximately 170,000 tons that are destined mainly for the United States, which is the main importer. The European Union ranks as the second largest importer due to the promotion of the nutritional benefits of this fruit [39].

Papaya pulp is a source of vitamin A, C and E with antioxidant action, of minerals such as magnesium and potassium and rich in pantothenic acid and folate. In addition to these nutrients, papaya contains an enzyme known as papain, widely used in the food industry and which is effective in improving intestinal transit and in the treatment of trauma, allergies and injuries [40].

### 3.5. Other Subtropical Fruits

#### 3.5.1. Custard Apple or Cherimoya

Custard apple or cherimoya (*Annona cherimola* M.) is a subtropical fruit that belongs to the *Annonaceae* family. It is a large fruit, with a green skin with round bumps, a sweet white pulp and small black seeds embedded in the pulp [41]. It is native to the Ecuadorian and Peruvian Andes, and is considered one of the most prized tropical fruits within the genus Annona spp. The *A. cherimola* plant requires a dry climate where there is not much rain and there are no sudden changes in temperature. Normally, it is a fruit that is consumed fresh, although different industrial products, such as juices, yogurt and creams, are also made [42]. The weight of custard apple ranges between 100 and 500 g, in which the weight of the peel reaches practically half the weight of the fruit [43]. 

It presents an excellent quality and commercial value, being cultivated mainly in the Andes, Europe and California. Spain is the world’s leading producer of cherimoya, cultivating more than 3600 ha in the south of the country, with 80% of the total cultivated on the Granada and Malaga coast, with the Campas and Fino de Jete cultivars being the most important varieties [44]. Regarding its nutritional value, custard apple has a high content of water, sugars and minerals, such as Ca and P. The main organic acids that we find in its composition are citric acid and malic acid and its high content of vitamin C [45].

Traditionally, the custard apple plant has been used for various diseases, mainly against digestive disorders and skin disorders, due to its antimicrobial effect attributed to the phenolic compounds present in it [46]. 

#### 3.5.2. Litchi

Litchi (*Litchi chinensis* S.) is a subtropical evergreen tree belonging to the family *Sapindaceae*. It has an indehiscent pericarp surrounding a palatable edible aril with a dark brown seed. China and Thailand account for most of the commercial litchi production. Its pulp is very high in nutritive value with carbohydrates, proteins, fibers, lipids, vitamins, amino acids and minerals.

#### 3.5.3. Passion Fruit

Passion fruit plant (*Passiflora edulis* S.), belonging to the largest genus of the botanical family *Passifloraceae*, is a Brazilian native plant now extensively cultivated in tropical and subtropical regions. These have recognized sedative effects, in addition to an impressive bioactive profile that allows several therapeutic applications: anxiolytic, anti-inflammatory, antioxidant, antimicrobial, antifungal, immunomodulator, etc. [47].

Passion fruit pulps are mainly used for juice production, and its seeds and peels, which comprise 50% of the whole fruit weight [48], are discarded. 

#### 3.5.4. Guava

Guava (*Psidium guajava* L.) belongs to the *Myrtaceae* family and *Psidium* genus and it is the largest of the minor tropical fruits in terms of production volume. A total of 6.5 million tons was estimated to have been produced in 2017, ranking India as the major guava producing country (56%), followed by Pakistan, Egypt, China and Brazil [49].

Guava fruit is a well-known source of vitamins (C), minerals, carotenoids (lycopene) and dietary fiber, and has high contents of phenolic compounds, predominantly composed of flavonoids (proanthocyanidins, flavanols, etc.), hydrolysable tannins (ellagic acid derivatives), phenolic acid, benzophenones, lignans, stilbenes, dihydrochalcones, etc. [50]. However, its main by-products (peels and seeds) are also sources of these compounds. In fact, the guava peel exhibits approximately 40% higher antioxidant activity than its pulp, and about 50% of the whole fruit is composed of peel (20%) and seed core (30%), commonly discarded during guava processing [51]. Thus, the interest for reusing these by-products in industries such as nutraceuticals, pharmaceuticals and cosmetics is increasing fast.

## 4. Obtainment of Enriched Bioactive Extracts from Tropical and Subtropical Fruit By-Products

Allegedly, nutritional substances and phytochemicals useful for the health and well-being of humans are found in tropical fruits, although mainly in an inedible fraction. To learn more about the benefits of these compounds, it is necessary to carry out the identification and characterization of the bioactive ingredients in them. The characterization of these compounds focuses primarily on the selection of efficient extraction methods, being the main objective to extract specific compounds from a complex matrix increasing the selectivity of the analytical method, the sensitivity of the assay and the number of target molecules and finally turning the substances into a more suitable way to be detected, separated and obtained. Therefore, the extraction process must be carried out carefully and optimally in order to reach the most reproducible method possible [52]. 

Commonly, the conventional extraction techniques for recovering high added value compounds from natural sources include Soxhlet, liquid-liquid hydro-distillation, maceration and mashing. However, a great deal of BACs is thermally unstable and can be either degraded or completely lost during the production of conventional extracts, in addition to the fact that these technologies involve high consumption of energy and often hazardous solvents, along with long extraction times, inefficient selectivity and low extraction yields [53]. In order to solve these problems, new sustainable extraction methods have emerged that seek both to improve the extraction performance and the quality of the extracts.

These alternative non-conventional extraction techniques are characterized by shorter extraction times, lower or even zero amounts of solvent required, with an environmentally friendly and sustainable, almost non-existent impact, straightforward in execution, with a decreased usage of energy and water. Solvent selection depends on the sample properties; in general, the most commonly used solvents for the recovery of these BACs are GRAS solvents that could be used in pharmaceutical and food products, e.g., water and ethanol or mixtures of them [2]. Within this category of less harmful extraction technologies, some have already been widely used: SFE, UAE, PLE, SWE and MAE, although the last two are not considered as the best option for BACs recovering for using temperature as an extraction parameter. Non-thermal technology-assisted extractions are considered as the most innovative and potentially useful methodologies for the extraction of BACs from fruits and other natural sources, since they operate at refrigerated or room temperatures, allowing heat-sensitive compound extraction. The extracts obtained are of a higher quality and with an improved biological activity. Besides those mentioned before, Pulsed Electric Fields-Assisted Extraction (PEFE) and High Pressure Processing/High Hydrostatic Pressure-Assisted Extraction (HPPE/HPAE) are two emergent techniques completely suitable for the obtainment of thermolabile BACs [54,55].

When both conventional and non-conventional extraction techniques are compared, differences are clearly appreciated: Trujillo-Mayol et al. (2019) performed simultaneous conventional and non-conventional extractions on four avocado peel samples, and compared extraction yields, phytochemical attributes and antioxidant activity of each one. Yield by maceration was lower than 8%, while UAE and MAE achieved more than 16%, and U-MAE (Ultrasound-Microwave-Assisted Extraction) more than 25% [56]. This trend was also seen on the total phenolic content (TPC): 270, 274, 281 and 257 mg gallic acid equivalent (GAE)/g dry extract (DE) for UAE, MAE, U-MAE and maceration, respectively. Kaur et al. (2021) also compared UAE with maceration extraction in mango peel samples, noting that TPC from maceration sample was excessively lower than UAE sample (20.5 versus 35.5 mg GAE/g, respectively) [57]. Khor et al. (2021) were able to conclude that *C. papaya* leaves supercritical CO_2_ (scCO_2_) extract improved phenolic content and total extraction yield, and exhibited stronger radical scavenging activity than conventional extracts [58]. Additionally, all the antioxidant activities of the HPP extract of longan fruit were higher compared to conventional extract of longan fruit [59].

On the other hand, unless this review is widely focused on phenolic compounds application, carotenoids importance cannot be ignored. These natural dietary products, broadly found in consumed products, such as tomatoes, carrots, citrus and derivatives, are known to be powerful antioxidant substances, playing an essential role in the reactions of ROS neutralization [60]. Some of them have been important as well for the their function as provitamin A, decreasing non-communicable diseases’ development risks and remarking their indisputable value in the context of nutricosmetic [61]. Therefore, and knowing that a great part of these compounds accumulates in the skin, carotenoids have an important role in the photoprotection against UV radiation, even enhancing the elasticity of the skin and its hydration [62]. Their extraction and isolation from fruit by-products have been deeply studied through the years, and constitutes an interesting process for developing novel products for different industries, taking advantage from wastes and reducing its negative impact on the ecosystem.

García-Mendoza et al. (2015) reported important antioxidant activity and high potential application in food, nutraceutical and pharmaceutical industries of the carotenoid-rich mango peel extract obtained by scCO_2_ and PLE, highlighting extraction selectivity, low temperature, short time and no pollution [63]. Similarly, Mercado-Mercado-Mercado et al. (2018) also showed how in vitro bioaccessibility of carotenoids from mango peel extracts improved using UAE as the extraction technology, improving carotenoids exposition for their release [64]. In contrast, Kapoor et al. (2020) performed two different extractions on guava seed: solvent extraction through Soxhlet method and scCO_2_, and extraction yields were found to be 12 and 10.5%, respectively. However, they did not perform antioxidant power evaluation on the scCO_2_ due to the lower yield; thus, no comparison of antioxidative capacity can be done [65].

Regarding the analytical techniques for the identification and quantification of BACs, the most widely used are online chromatography coupled with mass spectrometry (MS) or the diode array detector (DAD). High-performance liquid chromatography-mass spectrometry (HPLC-MS) and gas chromatography-mass spectrometry (GC-MS) are the analytical strategies used in food analysis, with HPLC being the preferred separation technique for the analysis of natural products. HPLC-MS is the main technique for the analysis of flavonoids with good sensitivity in ESI systems [66]. In particular, MS is often used in HPLC analysis to obtain high structural information and increase selectivity after HPLC-DAD screening [52]. 

In Table 1, more examples of bioactives extraction from tropical fruit by-products, and some analytical technologies applied for their identification and characterization, are shown.

Taking into account the increasing awareness on environmental problems, the growing interest in a circular economy and the revalorization of food by-products, the selection of a suitable environmentally friendly extraction technology which allows the recovery and sustainability of target analytes is indispensable. Thus, the non-conventional green techniques aforementioned are the perfect choice for the extraction of bioactive ingredients from these fruits, preserving sustainability and development. Part of the importance of these methods also relies on their optimization and future possibilities, using different solvents with specific natures, combining processes to promote extraction parameters, scaling to operational industries, etc., without compromising the quality of the final extracts. On the other hand, and knowing that bio-residues constitute interesting sources of these biologically active substances, to deepen knowledge of this field is of the utmost importance.

However, cosmeceuticals not only have the ability to provide benefits in the characteristics of the skin, but also work it from the inside depending on the presence of the natural active ingredient and its accessibility. Antioxidants are characterized by being unstable in environmental conditions or having low or no penetration because not all bioactive compounds have similar affinity for different layers of the skin [96]. Thus, in order to improve their topic action, these compounds need to be included into a vehicle. This will increase the bioavailability and penetration of the active ingredient in the deepest layers of the skin, in addition to providing stability and protection. An effective way to transport these compounds are liposomes, microscopic bilayer lipid vesicles that have been shown to be very useful in protecting assets. These liposomes, in addition to conferring stability and protection against external factors (light, temperature, pH and oxidation) that cause the degradation of these compounds, will provide numerous advantages, such as greater bioavailability, penetration and efficacy, biocompatibility, low toxicity and a good resizing ability. However, the application and manufacture of liposomes in cosmeceuticals presents a number of challenges, i.e., the decrease in the stability over time of the liposomes, the difficulty in encapsulation techniques and their efficiency and the limitations in skin permeability [97,98]. For this reason, further research on new techniques is needed to improve the quality of liposomes and their production at an industrial level.

## 5. Therapeutic Targets of Cosmeceuticals from Tropical Fruit By-Products Related to Skin Health

To understand the polyphenol’s importance as an active ingredient which targets the skin, it is of huge interest to detail the basic skin structure. Skin is the largest living organ of the human body, representing the biological barrier between the body and the external environmental conditions. Anatomically, the skin is composed of the epidermis, with structural and functional proteins, lipids and the top-most layer of cornified mature keratinocytes making up the stratum corneum, the dermis underlying the epidermis, which comprises mainly fibroblasts, responsible for the production of another component, extracellular matrix (ECM), and protein fibers composed of collagen and elastin, and subcutaneous tissue, which contains plenty of blood vessels and provides insulation [99,100].

The fibroblast is the characteristic and most abundant cell type of the connective tissue proper, and their primary function is to maintain the integrity of the connective tissue and to create a structural scaffold (extracellular matrix) for tissues and organs [101]. ECM is composed essentially of matrix metalloproteases (MMPs), ubiquitous endopeptidases playing a key role in skin physiological and pathological phenomena and collagen, elastin and elastic fibers, all essential ingredients to provide strength to muscles, tendons and joints [102]. Collagen accounts for about 80% of dry skin weight and is responsible for the tensile strength and maintaining flexibility of the skin. Elastin fibers, on the other hand, provide elasticity to the skin. There are hydrophobic molecules in the skin that prevent the loss of moisture, such as cholesterol, ceramides and fatty acids, but also hygroscopic amino acids derived from the degradation of filaggrin or hyaluronic acid (HA). The last one helps to retain moisture of the skin as well as its structure and elasticity, and is also involved in rapid tissue proliferation, regeneration and repair [100,103].

As we age, our body produces less collagen and elastin, which plumps our skin and makes it lose its elasticity [104]. The changes in the skin are among the most visible signs of aging, which include wrinkles, sagging skin, age spots and dryness, and also fat loss, making the skin lose its natural smoothness [104,105]. Moreover, there are other phenomena mainly caused by extrinsic factors which promote skin impairment and tissue damage, or even more hazardous issues at a cellular and genetic level. Therefore, the possibility of implicating the therapeutic nature of polyphenols to satisfy these therapeutic targets and even be able to revalorize some tropical by-products is exposed.

The main targets to be treated in order to achieve skin health are shown in the next sections.

### 5.1. Oxidative Stress and Skin Aging

Skin aging is a complex biological process accompanied by phenotypic changes in cutaneous cells, with structural and functional changes in extracellular matrix components. Although the underlying mechanism of skin aging is not yet completely understood, multiple pathways have been illustrated which have been speculated to be responsible for skin aging (changes in DNA repair and stability, mitochondrial function, cell cycle, apoptosis and cellular metabolism) [104]. Oxidative stress in the skin greatly influences this skin-aging process. It is a crucial issue in biology and is caused by time-dependent internal and extrinsic factors, such as ultraviolet (UV) light [106]. This process can be defined as a natural phenomenon, resulting from the disruption of the redox equilibrium, with pro-oxidants overcoming antioxidants, which can eventually result in cell or tissue damage [107]. Additionally, a disturbance of the redox equilibrium could be produced by excessive concentrations of antioxidant species, which could lead to the same disruptive state of the body. In this sense, it is important to explore the mechanisms of action of each species in order to optimize the safe dosage [108,109].

As the first interface between the environment and the immune system, the skin is constantly exposed to both exogenous and endogenous factors which can enhance oxidative stress and skin aging, and cause cutaneous damages and diseases as skin cancer. Although under normal conditions the endogenic antioxidant system of the skin (formed by enzymes such as superoxide dismutase (SOD) and catalase) is very effective, oxidative stress diminish its effectiveness [110]. Although the antioxidant role of SOD has been known and studied for years, since its capacity of destroying free radicals such as superoxide by converting it into hydrogen peroxide H_2_O_2_ [111], a considerable increase in the concentration of this signaling molecule can lead to highly reactive species that are toxic to the organism, such as OH· or ROO· [112,113]. This imbalance means that the anti-oxidative power of SOD may be negligible [114].

The main cause of oxidative stress is the overproduction of reactive oxygen species (ROS), which includes any chemical species with one or more unpaired electrons in its last layer, and comprises radical species such as superoxide (O_2_), hydroxyl (HO) and peroxyl (ROO·); and nonradical species such as hydrogen peroxide (H_2_O_2_) and singlet oxygen (^1^O_2_) [115]. ROS is considered one of the most important factors related to aging [116]. However, certain studies establish that mitochondrial ROS could have beneficial effects on the body such as cytoprotection, anti-inflammatory effects and prolongation of longevity. In any case, there is no a clear reason to establish that the presence of ROS implies aging, but this oxidative stress could be a consequence of aging [117].

In normal conditions (intrinsic aging phenomena), they are produced in our body during oxidative phosphorylation in mitochondria (respiration). Subsequently, they develop important roles in cell signaling phenomena and maintenance of skin function, and then are inactivated by the cellular antioxidant defense mechanism [101,104]. However, when the body is under pathological conditions (chronic wounds) or subjected to UV radiation, pollutants, stress and inflammation (extrinsic pro-aging factors), the oxidative stress triggers cellular damage pathways, causing senescence of cells through the generation of harmful ROS levels. This overproduction can derive in DNA and RNA damage, protein denaturation and some physiological tissue reactions, including lipid peroxidation and inflammation [9], contributing to adverse effects on the skin, expressed as erythema, edema, wrinkling, photoaging, hypersensitivity and photocarcinogenesis [116]. 

On the other hand, excessive ROS presence also stimulates matrix metalloproteinases (MMP) activation, as well as activating enzymes such as collagenase, elastase, tyrosinase and hyaluronidase, resulting in the degradation of collagen and elastin, damage to the dermal connective tissue and consequent premature skin aging through enzymatic degradation [115]. This event starts with a massive degradation of elastin material, called elastosis [103], with deposition of elastic fibers and collagen degeneration observed in photoaged dermis. Irregular and disorganized collagen provoke the weakening of the basal membrane separating the dermis and epidermis. Dermal fibroblasts, which make precursor molecules called procollagen, later converted into collagen, collapse through this mechanism due to the accumulation of degraded collagen fibers that prohibit construction of a healthy collagen matrix, causing an imbalance between synthesis and degradation [102]. 

There are also some pro-inflammatory cytokines involved in these physiological processes, since ROS and the induction of matrix metalloproteinases lead to its activation through several signal transduction pathways related to growth, differentiation, senescence and connective tissue degradation [101]. ROS initiate a chain reaction of lipid peroxidation in the cell membranes and intrude into the signal transduction pathways that are involved in the expression of genes. Then, the formation of inflammatory mediators, such as nitric oxide (NO) or cyclooxygenase-2, and proinflammatory cytokines, such as tumor necrosis factor (TNF-α), is promoted. For example, TNF-α is a cytokine produced in order to initiate a cascade of cytokines to recruit macrophages and neutrophils to a site of infection. All these intermediates are able to induce the collagen degradation by promoting apoptosis in dermal fibroblasts as well as enhance the expression of MMP and prevent the expression of procollagen [102,110]. The hyaluronic acid level present in the dermis is also diminished through intrinsic and extrinsic aging.

Therefore, those compounds which exert inhibitory actions against collagenase, elastase and hyaluronidase will have an effect of maintenance of ECM homeostasis and anti-aging properties for the skin, attenuating the loss of cutaneous elasticity and structure. Fibroblasts preservation is another important target for skin health, since it is responsible for the formation of the entire ECM and skin proteins, and they are an usual target by ROS and oxidative stress [2,100]. Hence, the desired bioactive profile should exert anti-collagenase, anti-hyaluronidase, anti-elastase and antioxidant activities, in order to detain the decrease in skin proteins levels and to be able to scavenge free radicals and alleviate the effect of ROS on fibroblasts.

### 5.2. Photoprotection

Although the sun is beneficial and essential for life, overexposing ourselves to sunlight might lead to detrimental health effects that can finally derive in skin cancer (90% are caused by sun) [104]. Previously, the influence that ultraviolet solar radiation exerts in premature skin aging has been exposed; nonetheless, a large amount of other serious health problems can be triggered: intracellular DNA damage, oxidative stress resulting from the photochromic generation of ROS and degradation of ECM components, such as collagen, fibronectin, elastin and proteoglycans, among others. All these physiological changes can cause multiple effects on the immune system and often generate or sustain UV-induced neoplasms with decreasing skin hydration and elasticity [118].

The UV sunlight irradiated to the Earth is divided into three distinct bands: UVC (280–100 nm), UVB (315–280 nm) and UVA (400–315 nm). UVC is practically absorbed by the ozone layer, so UVB and UVA rays do the most damage to the skin. UVB radiation consists of short wave and low penetration, responsible for damage that results in sunburn (erythema and edema) and oxidative stress induction, besides being a highly genotoxic agent [119]. UVB photons absorption can be led to disruption of DNA that can initiate photocarcinogenesis. However, it is less penetrating than UVA and only 5% gets the epidermal basal cell layer, which affects mainly the proliferation, differentiation and metabolism of these cells. UVA reaches basal dermis where the majority of skin cancers occurs; therefore, UVA is the most pro-cancerous radiation [120].

Photoaging is a combined difficulty, where a deterioration of biological functions and stress management is provoked to a large degree by UV and UV-generated ROS. UV acts via the activation of cell signal transductions, such as Mitogen-Activated Protein Kinases (MAPK) or Jun N-Terminal Kinase (JNK), as well as increase expression of NF-kB and activating proteins (AP-1), which finally leads to a promotion of MMPs generation and the disruption and degradation of ECM components. On the other hand, both UV and ROS also provoke erythema on skin (sunburn). Through NF-kB activation, this process derives in a stimulation of pro-inflammatory gene transcription and production of cytokines, such as interleukins or TNF-α, finally leading to a vicious inflammatory state cycle [107,120].

Sunscreen agents protect the skin by minimizing the damaging effects of harmful UV radiation from the sun. Sunblocks are the products which protect against UVB rays, while sunscreen protects against UVA rays [104]. When sunscreen is applied only 55% of free radical formation is reduced, but inserting antioxidants in sunscreen results in a radical scavenging improvement. However, there are some noteworthy factors in its formulation that can limit its application and beneficial purpose, such as the stability of antioxidants in the medium, penetration of the antioxidants into the epidermis (for instance, UV filters need to remain on the surface) or the mechanism of action, based on differences between inorganic blockers and organic absorbers [104].

As mentioned above, UV irradiation indirectly induces the expression of matrix metalloproteinases in the fibroblasts; especially MMP-1, which is mainly responsible for the degradation of the dermal collagen in photoaging. Thus, this contributes substantially to the connective tissue damage and wrinkling, one characteristic feature of photoaging. Additionally, irradiated keratinocytes promoted even more MMP-1 generation; thus, cosmetic ingredients are an ideal treatment because of their topical application.

### 5.3. Other Targets

#### 5.3.1. Wound Healing

Wounds are defined as a disruption of the normal anatomical structure and functional integrity of living tissue, and they can be classified as incisions, lacerations, abrasions, contusions, ulcers and burns [100]. In response to damage, the tissue undergoes a complicated process of healing.

Wound healing is the process of repair that follows injury to the skin and other soft tissues. Following injury, the healing cascade begins and an inflammatory response occurs (cytokines or growth factors). Then, the cells below the dermis begin to increase collagen production: there is a deposition of a fibrin clot at the site of injury, which serves as a provisional matrix and sets the stage for the subsequent epithelial tissue regeneration. Finally, epithelial cells crawl across the wound, which is contracted from each edge and undergoes contraction [107,121]. The efficiency of the wound healing process decreases with advancing age.

There are several stages to the process of wound healing: inflammation, granulation tissue formation, epithelization, collagen synthesis and tissue remodeling. Characterization for each phase includes presence of leukocytes, angiogenesis, protein synthesis and deposition, wound contraction and scar formation. Impaired healing by failure to progress through these stages derive in pathologic inflammation and chronic wounds, usually associated with ischemia, diabetes mellitus and pressure. In these cases, although minimal ROS prevents infections, the production exceeds average levels in response to NADPH oxidase 2 (NOX_2_) activation in macrophages and neutrophils during the inflammatory phase of the healing, contributing to high oxidative stress that leads to the wound not healing [107]. Despite this, inflammation is essential to prevent infection and stimulate angiogenesis and matrix deposition, creating cytokines and angiogenic factors at early stages of the wound, while prolonged pathological inflammation causes delayed wound healing and fibrosis. This means that inflammation is crucial for recovery, but not the entire process [122].

Afterwards, all mentioned above, collagen is needed to repair the defect and restore anatomic structure and function of the skin wound. Herein, alternative products such as phenolics could alleviate such damages if they were used as active ingredients. 

#### 5.3.2. Hyperpigmentation

Skin pigmentation is a complex process involving the synthesis of melanin and the proficiency of melanocytes cells. Melanin, the principal molecular determinant of skin color, provides protection by limiting the absorption of UV radiation by approximately 50–75% and scavenging ROS. These molecules are deposited once they are synthesized by specific organelles known as melanosomes, which undergo a maturation process followed by their transfer to keratinocytes [100,123]. 

In human beings, tyrosinase catalyzes both the hydroxylation of mono and the oxidation of diphenolase, both melanin precursors in its synthesis, and thus, they will protect the skin from the detrimental effects of UV radiation. Nevertheless, an excessive production is responsible for skin hyper-pigmentation, as in the case of melasma, freckles, ephelides and senile lentigines, which are important targets for skin-whitening agents [124]. Hyperpigmentation is an abnormal darkening of the skin mostly derived from excessive melanin production. It is typical of skin disorders, including melasma associated with pregnancy or age, freckles and photoaging, age spots, actinic keratosis, malignant melanoma and even neurodegenerative diseases such as Parkinson.

The basic mechanism of skin darkening starts with UV exposure and the consequent stimulation of α-MSH, a peptide hormone which promotes melanocytes production. This compound, once bound to specific active areas expressed on melanocyte surfaces, induces melanogenesis via multiple signaling pathways. For instance, MITF is a key transcription factor regulating the transcription of melanogenic enzymes, especially tyrosinase. Different products and pro-inflammatory cytokines from this cascades induces melanogenesis with lack of any control, resulting in DNA damage [125].

Various strategies have been developed to contrast skin hyperpigmentation, including the inhibition of melanin cession to keratinocytes, the speed-up of turnover in the epidermal layer, and the use of anti-inflammatory treatments. However, given the importance of tyrosinase in melanin synthesis, its inhibition is the most commonly pursued skin-whitening option [123]. In this context, plant-based and naturally derived skin-whitening agents have been examined for their biological activities and safety. 

#### 5.3.3. Skin Cancer

Skin cancer is a result of several mutations in cancer-related genes, including proto-oncogenes and tumor suppressors in skin cells, which causes an imbalance in cell homeostasis and excessive cutaneous cell proliferation. Based on the cell type, cutaneous melanoma and non-melanoma are the main classes of skin cancer. The latter originates from keratinocytes of the epidermis and is divided in two main subdivisions, which are basal cell carcinoma (BCC) and squamous cell carcinoma (SCC). Melanoma, originating from melanocytes at the deepest layer of epidermis, has the lowest prevalence rate, but the worst prognosis, and is responsible for the 80% of mortality from skin cancer [126].

Cellular processes, such as gene expression, cell cycle progression, development of proliferation and migration, are considered key factors in cancer regulation and prevention. Polyphenols may prevent cancer initiation (cytoprotective effect), relapse or its progression and metastasis to distant organs (cytotoxic effect). The cytoprotective property of polyphenols is generally attributed to their antioxidant activity, while the actual anticancer efficacy is due to antioxidant-independent mechanisms, including their pro-oxidant action. Therefore, polyphenols may produce antioxidant effects in normal cells, while inducing pro-oxidant damage in cancer cells. With respect to this pro-oxidant activity, polyphenols are able to generate ROS in cancer cells that lead to induction of apoptosis, suppression of cell cycle and downregulation of proliferation through the modulation of several signaling pathways [126,127].

#### 5.3.4. Acne

*Acne vulgaris* is a chronic inflammatory skin disease affecting approximately 85% of the population at some point in life, especially during puberty. It is characterized by the presence of papules, cysts and nodules. Acne is caused by multiple factors, including the stimulation of sebaceous gland activity, follicular hyperkeratinization, hormonal imbalance and inflammation caused by stimulation of innate immune system, primarily because external bacterial infection by Propionibacterium acnes and Staphylococcus epidermidis [100].

UV radiation induce remarkable amounts of oxidized lipids, triglyceride hydroperoxides and cholesterol hydroperoxides generation, leading to increase sebum secretion [107]. *P. acnes*, which grows using this sebum as its anaerobic environment, is denoted as the predominant bacterium due to its unique immunomodulatory effect: skin macrophages are directly induced by *P. acnes* heat-shock proteins to produce proinflammatory cytokines including neutrophils and interleukins. This last type mainly stimulates neutrophils migration, leading to acne lesion and pus formation. Subsequently, neutrophils generate ROS for killing the bacteria, but excessive production derive in ROS leakage within extracellular space, which destroys follicular epithelium and accelerates the progression of inflammatory responses [128].

According to this, identification of new pharmacological interventions for its management seems to be necessary. Tests employed for the discovery of ingredients with potential anti-acne activities rely on the antibacterial and anti-inflammatory properties of the test substance. Therefore, plant-derived molecules, mainly phenolics and polyphenols, are ideal choices since they have been proved to exert important antibacterial and anti-inflammatory functions. Several molecular mechanisms have been found to have effects on acne vulgaris, including anti-inflammatory, antioxidant, antibacterial, immunomodulatory, reducing sebum production, decreasing lipogenesis and antiandrogenic activities [129].

## 6. Bioactive Content and Therapeutical Application of Tropical and Subtropical Fruit By-Products

**Figure 2:**
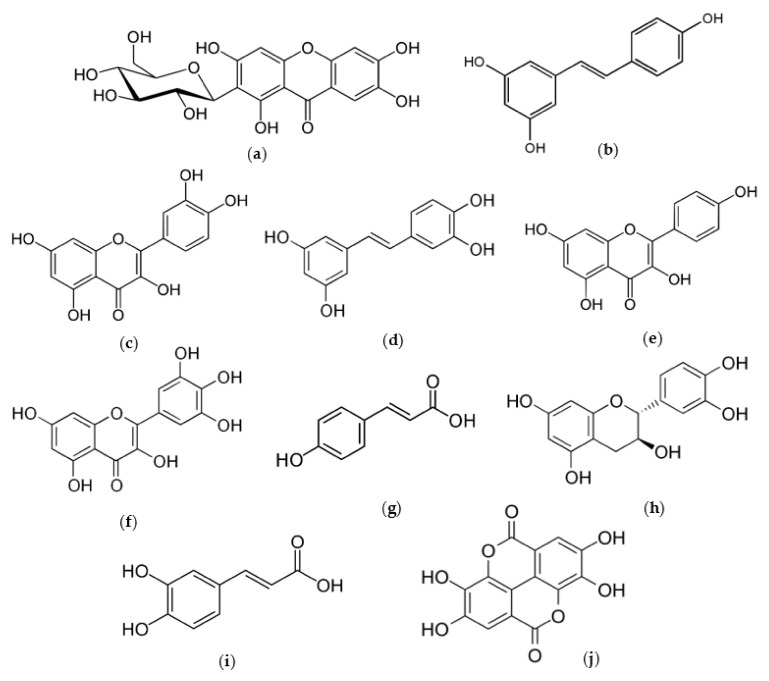
Some highlighted phenolic compounds: mangiferin (**a**), resveratrol (**b**), quercetin (**c**), piceatannol (**d**), kaempferol (**e**), myricetin (**f**), p-coumaric acid (**g**), catequin (**h**), caffeic acid (**i**) and ellagic acid (**j**).

There are a great number of studies that demonstrate the phenolic wealth, and the anti-aging and antioxidant effect of tropical fruits by-products on skin, as well as how they can be exploited from a cosmetical point of view (Table 2). Both in vivo and in vitro assays have been performed in those papers using different parts of mango, avocado, custard apple, pineapple, lichi, guava, passion fruit and papaya. Phytochemicals (Figure 2) in all these fruits are known thanks to their effectiveness as free radical scavengers and metal ion chelators, being able to reduce pro-inflammatory factors and to exert a wide range of biological activities: photoprotection, anti-tumorous, cytotoxic, cutaneous aging-related skin enzymes modulator, etc. [109,127]. After reviewing, it can be concluded the high-added value these by-products harbor is mostly due to its phenolic wealth.

### 6.1. Mango (Mangifera indica L.)

Mango by-products give an added richness to the fruit. In the case of the kernel, it far exceeds the lipid content of the pulp, allowing high value oil to be obtained. It is also composed of important antioxidant minerals, such as selenium, zinc, copper, manganese and potassium, a large number of polyphenols, such as mangiferin, quercetin and kaempferol, and phenolic acids, such as gallic, caffeic, coumaric and ellagic. In the case of the skin, it exceeds in quantity the phytochemical level of the pulp and the kernel, being the part of the plant that has the highest content of mangiferin, which is the majority compound [130]. Other majority compounds included in peels are gallotannins such as quercetin derivatives [24].

These BACs, which both edible and inedible mango parts harbor, are protagonists of multiple beneficial effects; thus, they are used in traditional medical systems for centuries as a treatment of a wide variety of human diseases [25]. Its pharmacological profile consists of anti-inflammatory, antihyperglycemic, antioxidant, antifungal, antiviral, hypotensive, hypolipidic, antiulcer, analgesic, antibiotic, antibacterial, hepatoprotective, neuroprotective, gastroprotective, immunomodulatory, antidiarrheic, anticancer and antitumoral activity [25], even as a prebiotic [131], closely related for nutraceutical purposes [2,20]. In this way, mango and its main residues are prepared to act by reducing cardiovascular risks, diabetes, obesity, neurological disorders or various cancers, among many others [2].

Much of the importance of this therapeutic value lies in the ability of these substances to reduce the level of oxidative stress by acting against free radicals and reactive oxygen species (ROS). This points to mango and its by-products as possible tools that give some added value to products from the food, pharmacological and cosmetic industries [22]. Great advances have been made in the latter in recent years, linking skin photoaging and collagen degradation with ROS formation due to exposure to UV light. Numerous studies have shown the cosmeceutical effect of mango, which is responsible for reducing collagen damage and loss (or even stimulating its synthesis) due to this exposure [132].

Cellular senescence is characteristic of aging, there is a permanent state of cell cycle arrest induced by cellular stress. During the aging process, senescent cells accumulate more and more in tissues, leading to a loss of tissue repair capacity due to cell cycle arrest in progenitor cells, producing pro-inflammatory molecules and ultimately contributing to development of various age-related diseases. Genetic evidence has shown that the elimination of senescent cells can delay aging and extend lifespan [133]. An example of a compound with senolytic power is quercetin, a compound present in mango and its by-products [24]. In 2015, quercetin was first discovered to be a senolytic agent that can effectively kill senescent human endothelial cells and mouse bone marrow-derived mesenchymal stem cells (BM-MSC) [134].

Poomanee et al. (2018) performed an in vitro investigation of anti-acne properties of mango kernel extracts. Final findings pointed it out as a promising active ingredient against acne primarily due to its notable antimicrobial and antioxidant activities, and its anti-inflammatory effect promoting inhibition on IL-8. Additionally, they demonstrated the ability to disrupt the bacterial cell membrane [128]. Ochocka et al. (2017) isolated mangiferin from leaves, peels and barks from mango tree and fruit, and reported its ability to penetrate skin and, once there, reduce oxidative stress blocking elastase and collagenase activities. Additionally, mangiferin’s safety and effectiveness made them consider its use in cosmetic and dermatological preparations [103].

### 6.2. Avocado (Persea americana L.)

Avocado peel and seed are high in bioactive phytochemicals such as phenolic acids, condensed tannins and flavonoids, including procyanidins, flavonols, hydroxybenzoic and hydroxycinnamic acids. These BACs have shown antioxidant and anti-inflammatory activity, which is related to their ability to eliminate free radicals responsible for oxidative and cellular stress [26]. Different studies establish that polyphenols are the most important BACs present in the avocado seed, which contains quinic, citric and malic acids and phenolic acids, mainly hydroxybenzoic acid, hydroxyphenyl acetic acids and hydroxycinnamic acid. Additionally, noteworthy is the presence in the seed of flavonoids, such as quercetin and catechins, and the high content of condensed tannins, such as procyanidins [77]. Thanks to the phytochemical characteristics of avocado, there are numerous studies that show that the avocado fruit, due to its high content of polyphenolic compounds, has antibacterial, antiallergic, platelet aggregation inhibitor, antihyperglycemic, antioxidant, anti-inflammatory and chemo-protective properties [76]. 

In the industrial processing of avocado to obtain different IV and V range products, the pulp is used while the seed and skin are discarded, giving rise to large amounts of by-products. These by-products produce economic losses and have an environmental impact due to their organic load. However, there is evidence that the skin and the seed may have even more BACs than the pulp and, therefore, have a more powerful antioxidant and anti-inflammatory activity [13]. That is why the extracts from these by-products can be used as bioactive ingredients in the pharmaceutical, cosmetic and food industries. There is evidence that the avocado seed and skin are a good source for cosmetic products [76]. Likewise, other studies indicate that the extracts obtained from avocado skin are of interest for the formulation of nutraceuticals with antioxidant and anti-aging activity [75].

The skin is the visible sign of the aging process and is frequently subjected to oxidative and inflammatory damage due to its exposure to UV radiation. The carotenoids together with lutein and zeaxanthin that are highly available in avocado help protect the skin from UV radiation damage and oxidative stress, thus having a photoprotective and anti-aging action [135]. Therefore, by-products from avocado would be a good option to obtain these BACs and their use in the production of cosmetics for facial skin.

Nayak et al. (2012) found that avocado leaf extract significantly increased the rate of wound contraction, epithelialization, weight of wound tissue and collagen synthesis. Additionally, its vasorelaxant action seems very helpful so as to the wound to be exposed to increased blood flow, thus being accompanied by the necessary inflammatory cells and factors that fight infection and debride the wound of devitalized tissue [121]. Rosenblant et al. (2011) proved that avocado seed ethanolic extract exerted anti-inflammatory and photoprotective activities, increasing cell viability and DNA repair and enhancing skin cancer protection [136]. Deuschle et al. (2019) also determined avocado leaves capacity to exert photoprotection against UVB radiation and promote antinociceptive effect, using the extract to prepare a *P. americana* gel which meant a successful treatment against skin lesions caused by radiation [119].

### 6.3. Pineapple (Ananas comosus L.)

The by-products of pineapple, formed almost entirely by the shell, core, stem and crown, are also very rich in fibers, pectins, cellulose and hemicelulose, as well as free sugars and a multitude of micronutrients of high biological value, such as vitamin C, minerals, polyphenols, phenolic acids and carotenoids (β-carotene) [34]. It is known for its antioxidant richness, with compounds such as myricetin, salicylic acid, tannic acid, trans cinnamic acid or p-coumaric acid [137]. Small amounts of ferulic and syringic acids promote this antioxidant and antimicrobial power [72]. In general, its nutritional composition makes these remains offer a significant bioactive profile supported by numerous studies: cytotoxic, antidiabetic, antihyperlipidemic, antinephrolithiatic, anticoagulant and anticancer, among others [35].

In recent years, bromelain has managed to be a molecule of high value thanks to its numerous biotechnological applications: beer clarification, gluten degradation in bakery, meat tenderization, improvement of the properties of cheese, anti-browning agent, alcohol production or its usefulness against chemical catalysts [36,138].

In addition, pineapple has properties and compounds with a very high cosmetic interest. Bromelain is used as an active ingredient in many cosmetic products. It is mainly part of products dedicated to skin care; it can be used to treat complications, such as wrinkles, acne, dry skin, burns or enemas. The procedure it performs is based on gently digesting and reducing the width of the protein layer of dead cells (*Stratum corneum*), encouraging its replacement with younger cells [32]. It is effective in the prevention and treatment of cellulite, has soothing effects on wounds caused by shaving and is able to decrease the intensity of spots on the skin [139]. On the other hand, ferulic acid is added as an active ingredient to numerous skin topics, as it has a very significant UV absorption capacity. By possessing a tyrosine-like structure, ferulic acid is believed to inhibit the formation of melanin through competitive inhibition with tyrosine. In addition, it provides in conjunction with vitamin C photoprotection activity of four to eight times greater against stimulated radiation damage, giving greater value to the pharmacological and cosmetic power of pineapple and its by-products [140,141].

The assay performed by Rahayu et al. (2017) used tacorin, a proteolytic extract from pineapple crown, core, butt, leaf and stem, to stimulate wound healing through a decrease of TNF-α expression. In this sense, tacorin is suggested to protect the progression of inflammation via suppression of TNF-α level, reduction of MMP-2 levels and enhancement of tumor growing factor (TGF-β; related to cell proliferation), which is related to its ability to promote fibroblast cell growth [142]. Additionally, Abbas et al. (2021) determined the antimicrobial activity of bromelain extracted from pineapple pulp, stem, peel, core and crown, turning it in a suitable substance to fight back acne and other similar conditions related to the presence of bacteria [143].

### 6.4. Papaya (Carica papaya L.)

*C. papaya* is also known for its numerous health benefits and properties due to its nutritional value and its content in BACs with nutraceutical characteristics [144]. Different parts of this plant are used as a treatment for various ailments, such as asthma, eczema, diabetes or ulcers, as well as for wound healing, cardiovascular diseases and cancer [145]. The agro-industrial waste of papaya, in particular the papaya seed, is a good source of antioxidant compounds for being rich in phenolic compounds, such as benzyl isothiocyanate, glucosinolates, tocopherols, B-cryptoxanthin, b-carotene and carotenoids [82]. In turn, the leaves from the papaya tree have a high content of dietary fiber and polyphenolic compounds, such as flavonoids (kaempferol and myricetin), alkaloids (carpaine, pseudocarpaine and dehydrocarpain I and II), saponins, proanthocyanins, tocopherol, lycopene and benzyl isothiocyanate [146].

There are numerous studies that have reported that the different parts of papaya have a powerful anti-inflammatory, immunomodulatory and antimicrobial activity. Other studies have indicated that papaya is also an excellent source of beta-carotene and, therefore, prevents damage caused by free radicals, thus having a good antioxidant capacity [147].

Kong et al. have researched how papaya fruit exerts this wound healing effect from a mechanistic point of view, finding potent antioxidant action of the papaya extract against H_2_O_2_-induced oxidative stress specifically on fibroblast cells was activated via radical scavenging, reduction of calcium ions influx into cytoplasm, reversal of oxidative stress-induced mitochondrial dysfunction and maintaining an oxidative balance inside the cells [107]. Moreover, photoprotective effects reported by Seo et al. (2020) using papaya leaf extract demonstrated that it suppressed some signaling pathways related to skin photodamage and prevented the degradation of procollagen [148].

The extracts from the seed and the skin of papaya may be of interest to the pharmaceutical and cosmetic industry as a source of bioactive ingredients to obtain products with added value. This would be an alternative for the efficient use of agro-industrial waste from papaya.

### 6.5. Other Subtropical Fruits

#### 6.5.1. *Annona cherimola* L.

Recently, it has been shown that different parts of the custard apple that are not consumed have a very interesting phytochemical profile, with a high content of polyphenols and alkaloids [81]. The seed, leaves and stem were reported as important sources of essential oils, flavonoids, alkaloids, saponins, acetogenins and phytosterols, among others, with nutritional, pharmaceutical and industrial interest. These compounds are characterized by their bioactivity, presenting antimicrobial, antiparasitic, antioxidant and antidiabetic activities [149]. Therefore, custard apple contains BACs with antioxidant properties that can contribute to the prevention of diseases associated with oxidative stress, such as cancer, atherosclerosis and neurodegenerative diseases. Other research [78] established that the custard apple peel is the richest part of the fruit in phenolic compounds, followed by the seeds and pulp. The custard apple peel exhibited a high content of catechins, epicatechin, procyanidin, hydroxytyrosol hexoxide, vanillic acid and phenylpropanoids.

Chai et al. (2017) used cherimoya pericarp proanthocyanins in order to evaluate their structure, anti-tyrosinase activity and mechanism of action. Finally, it was proven that these phenolics were a good choice to exert skin whitening effects. Study on the mechanism proposed a chelate formation between proanthocyanins and copper irons located in the active site of tyrosinase [10]. De Moraes et al. (2021) also proved the high antioxidant and antiradical capacity of atemoya pulp, peel and seed extract, showing its ability to scavenge free radicals and ROS, reduce oxidative stress and prevent cell damage [150].

#### 6.5.2. *Litchi chinensis* S.

Pericarp and seed are estimated to contain 30% of the dry weight of the entire fruit, and harbor a significant number of BACs, mainly flavonoids, proanthocyanidins and anthocyanins, responsible for its antioxidant, antimicrobial, anti-diabetes, antiviral, anti-tyrosinase, anti-inflammatory, immunomodulatory, anti-cancer, neuroprotective and cardioprotective activity [151]. Thus, lychee can be used as an easily accessible source of natural antioxidants from their by-products and/or as a possible agent in the cosmetic and pharmaceutical industry [152]. In fact, using ethyl acetate as a solvent, litchi extracts were proved to be useful in preventing photooxidative cell damage from UVBs. Its antioxidative and antityrosinase ability makes litchi wastes excellent cosmetic active ingredients [153].

In research by Lourith et al. (2020), who prepared a formulation with lychee extract for skin hyperpigmentation and aging treatment, the cosmetic was demonstrated to suppress cellular melanin production via tyrosinase and tyrosinase-related protein inhibitory mechanisms, with the extract being more potent than the standard [154]. Thiesen et al. (2017) also proved that litchi extracts from leaves have great potential to be used as an alternative to synthetic filters because of their ability to improve antioxidant endogenous proteins such as SOD. This is important for the avoidance of skin cancer and others undesired UV radiation effects [155].

#### 6.5.3. *Passiflora edulis* S.

Seeds and peels from passion fruit have an important nutritional value: the seeds have a high lipid content while the pericarp are sources of fiber and essential minerals, as well as an important level of functional active compounds, such as polyphenols and flavonoids [156]. 

Maruki-Uchida et al. (2013) demonstrated that passion fruit seed extract, and mostly piceatannol as the principal active ingredient in the seed, increased the intracellular glutathione (GSH) levels in keratinocytes and suppressed the MMP-1 induction in the fibroblasts, even without any irradiation [157]. In this sense, from a cosmetic point of view, both passion fruit and by-products extracts are extremely effective due to their anti-aging potential: resveratrol and piceatannol, representative phytoalexins conforming the fruit matrix, are known for skin lightening effects, preventing skin damage, inhibiting melanin synthesis and promoting collagen production [157,158]. On the other hand, Jusuf et al. (2020) determined passion fruit seed extract capacity to enhance prevention and therapeutic activity against *P. acnes*, exerting a potential antibacterial effect and, thus, reducing acne disorder [159].

The stilbene resveratrol previously mentioned has a high anti-aging potential, as it has been shown to be a possible direct activator of sirtuin 1 (SIRT1), capable of mimicking the benefits of caloric restriction in multiple organisms [158]. Different transgenic mouse models clearly illustrate that AMP-activated protein kinase (AMPK) and SIRT1 are key mediators of the metabolic health actions elicited by resveratrol [160]. A recent study by Park and his colleagues provided evidence, suggesting that direct inhibition of phosphodiesterase (PDE) could be the reason for the metabolic benefits exerted by resveratrol. The direct inhibition of PDE 1, 3 and 4 by resveratrol, at IC50 between 6 and 14 μM, would lead to the activation of protein kinase A (PKA) and the phosphorylation of AMPK in a Ca^2+^-dependent manner, greater availability of NAD^+^ and, finally, higher activity of SIRT1 [161]. Thus, this elucidates one of the possible activation mechanisms of SIRT1.

#### 6.5.4. *Psidium guajava* L.

Guava seed and peel are predominantly composed of proanthocyanidins, flavanols, ellagic acid derivatives, phenolic acid, benzophenones, lignans and stilbenes, among others [50]. Within its bioactive profile, guava by-products show compelling activities: anticancer, antihyperglycaemic, hypolipidemic, hepatoprotective, antimicrobial, cytotoxic, antioxidant, anti-inflammatory, etc. [51,162]. 

From a cosmetic point of view, there are examples of phytocosmetics using guava by-products that presented a higher photoprotection in synergy with UV filter from the reference formulation [163]. Pongsakornpaisan et al. (2018) treated acne not by directly attacking the causative bacteria, but instead reduced sebum levels using guava leaf extract to create a guava toner. Finally, they observed a significant suppression of sebum secretion [164]. Yasukawa et al. (2015) also showed the inhibitory effect that guava leaves exerted on inflammation in ear edemas, even alleviating the tumoral growing and other skin injuries in rats [165].

## 7. Conclusions

The enhanced consumption of tropical fruits in recent years, together with the production of fifth-range foods, has generated an increase in the production of by-products from this type of fruit. This fact has aroused growing interest in the revaluation of these by-products.

Phenolic compounds, in addition to being widely present in the pulp of tropical fruits, are also found in their by-products; thus, due to the important bioactive potential they present, the extraction of the compounds present in fruit residues for later application has been the object of a number of studies. In this sense, there is a growing interest in green extraction techniques due to the benefits discussed above.

Thus, some of the most widely used green extraction techniques are SFE, UAE, MAE and PLE. SFE has been used for the extraction of BACs from by-products of custard apple and papaya. UAE for the extraction of phenolic compounds from mango, avocado, custard apple, papaya and guava by-products. MAE has been used for the extraction of BACs from mango, avocado, papaya and passion fruit by-products. Finally, the PLE technique has been used for the extraction of these compounds in avocado by-products.

The BACs present in the by-products of these types of tropical fruits have been shown to exert a therapeutic effect against different targets to maintain proper skin health. Thus, in relation to oxidative stress and skin aging, the BACs present in by-products of mango, avocado, custard apple, pineapple, lychee, guava, passion fruit and papaya have been shown by in vitro and in vivo tests to be effective as free radical scavengers and metal ion chelators. Another benefit for the skin in which these compounds have been shown to exert a bioactive effect is photoprotection, among which the compounds present in mango pulp, avocado seeds and leaves, papaya leaves, leaves of lychee, the seed of the passion fruit and the pulp, the skin and the seed of the guava. Other targets related to the protective effect of the skin is the power of these compounds to improve wound healing, among which compounds present in mango oil, avocado pulp, pineapple stem and papaya seed stand out. Against the hyperpigmentation of the skin, the power of the compounds presents in the mango seed, in the avocado seed and the pericarp of custard apple stands out. Finally, there are also tropical fruits that have been shown to have an effect against acne, for example the compounds present in the mango seed, those present in the pulp, stem, peel, core and crown of pineapple and those present in the seed of the passion fruit.

After the bibliographic review carried out in the present work, the enormous potential of the revaluation of by-products from tropical fruits can be observed due to their high content of BACs. This revaluation has grown enormously in recent years from the point of view of the pharmaceutical and cosmetic industry in order to develop cosmeceuticals to alleviate skin pathologies. In this sense, these by-products of tropical fruits are sources of BACs that have been shown to deal with different skin-related pathologies.

**Table 2:** Properties of cosmeceuticals made with tropical fruit by-products.

Fruit	Wastes	Extract Type	Assay	Activity	Effect	Results	Ref.
Mango	Seed	95% ethanolic shook, refluxed or using acid hydrolysis extracts	In vitro	Anti-tyrosinase (skin lighter)	In vitro antioxidant activity (DPPH, chelating activity, etc.) and inhibition of tyrosinase; in vivo acute skin irritation tests	TPC: 286–90 mg GAE/g ID_50_: 4.13–7.45 mg/mL Competitive inhibitory effects of MSE due to binding with copper (metal at the center of the active site of tyrosinase). No irritating on skin.	[166,167]
		Two different 95% ethanolic extracts from *Kaew* and *Choke* species	In vitro	Anti-wrinkle and anti-hyperpigmentation	Antioxidant activity, inhibition of tyrosinase and hyaluronidase and skin irritation on in vivo clinical tests	TPC: 138.71/170.63 mg GAE/g DPPH: 197/254.64 mg Trolox/g Tyrosinase: 20.64/19.86 μg/mL (IC_50_) Hyaluronidase: 47.61/37.28 μg/mL (IC_50_) Cosmetic cream 1% MSE physically stable and safe for humans.	[168]
		Ethanolic extract	In vitro	Anti-acne	Antimicrobial activity against *P. acnes, S. aureus* and *S. epidermidis*	*P. acnes*: 1.56 mg/mL *S. aureus* and *S. epidermidis*: 3.13 mg/mL	[128]
		Mango oil	In vivo	Anti-wrinkle, wound healing, emollient effect	Antiseptic, healing, soothing and cooling activities, wound repair and closure, minimum scar formation	Foot-care cream provided emolliency, which rebuilt the skin’s protective lipid barrier and actively replenished moisture. No irritation or sensitivity after its application. Re-epithelization of wounds. Scars free of marks or lesions.	[169]
		Hydroethanolic extract	In vitro	Anti-aging and anti-hyperpigmentation	Antioxidant and anti-enzymatic activity; anti-inflammatory and cytotoxic activities, ability to prevent DNA damage and to inhibit NO	TPC: 800 mg GAE/g; DPPH: 7.35 mg Tr./g Tyrosinase: 1.09 μg/mL (IC_50_) MMPases 2 and 9: 0.46 and 0.11 mg/mL (IC_50_) HAase: 0.2 mg/mL (IC_50_) Depigmenting and moisturizing extract for the prevention of atypical brown spot and skin dehydration.	[9]
	Leaves	Hydroethanolic extract	In vitro	Anti-tyrosinase (skin lighter)	In vitro antioxidant activity (DPPH) and inhibition of tyrosinase	TPC: (40.00 ± 0.84) mg GAE/g DPPH: 7.35 mg Trolox/g Tyrosinase: (17.62 ± 1.26) μg/mL (IC_50_)	[170]
	Pulp	Aqueous extract	In vivo	Photoprotection	UVB protection and anti-photoaging activity	100 mg of mango extract/kg body weight per day inhibited UVB-induced increases in skin thickness, wrinkle formation and collagen fiber loss.	[120]
		Ethanolic and aqueous extracts	In vitro	Photoprotection	UVB protection	TPC Ethanol: (3.04 ± 2.52) mg GAE/g TPC Aqueous: (3.22 ± 0.11) mg GAE/g 250 μg/mL of aqueous extract was able to significantly reduce cellular apoptosis.	[171]
	Leaves, peel and bark	Mangiferin	In vitro and ex vivo	Anti-elastase and anti-collagenase	Anti-aging activity	Mangiferin’s ability to permeate through the stratum corneum barrier and to the living skin layers verified. Elastase: (139.64 ± 9.34) μM (IC_50_) Collagenase: (253.57 ± 7.56) μM (IC_50_)	[103]
Avocado	Seed	Ethanolic extract (polyhydric fatty alcohols)	In vitro	Anti-inflammatory and photoprotection	Increased cell viability, decreased sunburn cells, improves DNA repair in and reduces UVB-induced IL-6 and PGE 2 production in keratinocytes	CPD (photoproducts) removal in cells treated with PFA at concentrations 1 and 5 μg/mL was 92.3% and 74.5%. Protective properties against UVB cytotoxicity in cultured keratinocytes and human skin explants.	[136]
		Ethyl acetate extract (catechin)	In vitro	Anti-hyperpigmentation	Tyrosinase inhibition	Skin lightening agents through inhibiting the tyrosinase action. IC50 < 100 ug/mL	[172]
	Leaves	Hydroethanolic extract in gel form at 10% (catechin, chlorogenic acid and rutin)	In vivo	Photoprotection	Reduced UVB radiation-induced mechanical allodynia in rats and presented an antinociceptive effect in a UVB radiation-induced burn model	Compounds in highest concentration: (+) Catechin (302.2 ± 4.9 μg/g), chlorogenic acid (130 ± 5.1 μg/g) and rutin (102.4 ± 0.9 μg/g). Successful treatment against skin lesions caused by UVB radiation with *P. americana* gel (3%).	[119]
	Pulp	Avocado oil (phytochemicals)	In vivo	Wound healing and anti-inflammatory	Significant increase of epithelial tissue from wounds and in tensile strength proportional to collagen deposition, and reduction of inflammatory cells in scar tissue	Promote collagen synthesis and decrease the number of inflammatory cells during the skin wound healing process.	[173]
		Ethanolic extract of avocado (100 mg/kg)	In vivo	Wound healing	Suppression of symptoms of induced atopic dermatitis, of serum levels of IgE, of histamine and of inflammatory cytokines (TNF-α and IL-6), NF-ΚB and caspase-1 in skins lesions similar to atopic dermatitis	Significant reduction in the symptoms of atopic dermatitis such as itching, erythema, edema and dryness in mice	[174]
	Seed and Peel	Ethanolic extract of avocado peels and seeds (epicatechin and procyanidin B2)	In vitro	Anti-oxidant and anti-inflammatory	HOCl, ROO^·^ and O_2_^·^ removal capacity, and suppression of TNF-α and NO release on RAW macrophages at a concentration of 10 ug/mL of extract	TPC APE: 120.3 mg/g and ASE: 59.2 mg/g. DPPH APE: (420.5 ± 23.2) μmol/g and ASE (464.9 ± 32.7) μmol/g. FRAP APE: (1881.4 ± 75.3) μmol Fe^2+^/g and ASE: (931.7 ± 65.6) μmol Fe^2+^/g. O_2_^·^ APE: (52 ± 5) μg/mL and ASE: (70 ± 2).	[26]
Pineapple	Stem	Tacorin	In vitro and in vivo (rats)	Wound healing	Decrease of wound area by reducing the expression of TNF-α, promoting the expression of TGF-β and maintaining the expression of MMP-2 on treated rats	Tacorin treatment (80 mg/kg body weight per day) increased cell viability promoting regeneration, proliferation, cell growth and maturation. Its wound healing activity is suggested to be related to its ability to promote fibroblast cell growth, important in the formation of granulation tissue that is needed for wound closure.	[142]
	Crown	Dialyzed extract	In vitro	Anti-collagenase activity	Tissue remodeling and wound healing process	Topical application of the leaf extract in soft tissue injuries and hematomas where the extract can prevent the microbes to invade the host through the wound.	[175]
	Pulp, stem, peel, core and crown	Dialyzed extract (Bromelain)	In vitro	Anti-acne	Antimicrobial activity against *S. aureus, P. acnes, E. coli, C. diphtheria* and *P. aeruginosa*	PPPE exhibited highest inhibitory effects. DPPH PPPE: 13.158 μg/mL (IC50). *P. acnes*: 30 μg/mL (PPPE MIC). *S. aureus*: 15 μg/mL (PPPE MIC).	[143]
Papaya	Seed	Ethanolic extracts	In vivo	Wound healing and antimicrobial	Significant collagen deposition and fibroblast activity, antimicrobial activity against *S. choleraesuis* and *S. aureus,* and high wound shrinkage.	Powerful healing, antimicrobial and anti-inflammatory activity. Concentration of 50, 100, 150, 200 and 150 mg/kg body weight for 13 days.	[11]
	Pulp	Hydroethanolic extract	In vitro	Anti-oxidant and anti-aging	H2O2 scavenging activity and free radical scavenging (DPPH)	Anti-aging and skin renewing activity. Concentration of 0.62 to 4.96 mg/mL and 50 to 400 μg/mL, respectively	[176]
		PBS extracts	In vivo	Anti-inflammatory and anti-oxidant	Significantly increased activity of SOD, catalase and glutathione peroxidase, and suppression of COX-2 activity	Important antioxidant, anti-inflammatory and healing effect on skin wounds. Concentration of 5 mg/mL.	[177]
	Leaves	Ethanolic extracts (caffeic acid and rutin)	In vitro	Photoprotection	Free radical scavenging (DPPH), decreased UVB-induced expression of MMP-1, MMP-3 and IL-6 and increased TGF-β1 and expression of procollagen mRNA	Possible agent for treating skin conditions and photoaging.	[148]
		Methanolic extracts	In vitro	Wound healing	Free radical removal (DPPH), migration and proliferation of new cells in the wound area and increased collagen synthesis	In vitro wound healing ability using human skin fibroblasts. Collagen synthesis: 12.5 μg/mL for 24 h. DPPH IC50: 0.377 mg/mL (UAE), 0.236 mg/mL (Reflux), 0.404 mg/mL (Agitation).	[12]
	Fermented preparation	Fermented papaya preparation	In vivo	Anti-aging, anti-oxidant and skin improvement	Improved hydration, elasticity and skin color, and increased expression of aquaporin-3, decreased levels of MDA and significantly increased levels of SOD and NO production	Significant improvement in skin hydration and elasticity. Double-blinded study in subjects: 9 gr per day for 90 days.	[178]
Custard Apple	Pericarp	Proanthocyanidin extract (catechin and epicatechin)	In vitro	Anti-hyperpigmentation	Tyrosinase inhibition	Prevent melanin build-up and serious skin conditions, such as melasma, freckles, age spots and actinic damage.	[10]
	Pulp, peel and seed	Ethanolic extracts	In vitro	Anti-oxidant	Antioxidant capacity by scavenging free radicals (ORAC, ABTS and FOLIN)	Eliminate free radicals, reduce oxidative stress and prevent cell damage.	[150]
	Leaves	Methanolic extracts	In vitro	Anti-hyperpigmentation	Inhibit α-MSH-induced melanogenesis in B16F10 cells and melanogenic-related enzymes, such as tyrosinase, TRP1 and TRP2	Dermatological anti-hyperpigmenting agent for the treatment of skin diseases. Concentration of 1.25 to 5 ng/mL.	[179]
Litchi	Leaves	Ethanolic 70%	In vitro	Photoprotection	Antioxidant and photochemoprotective activity against H_2_O_2_, UVA and UVB; photoprotector agent against UV-induced DNA damage	No irritating agent/do not promote cytotoxicity in fibroblasts (0.1–100 μg/mL). Preserved cell viability (75% UVA 0.1 μg/mL–120% UVB 100 μg/mL) and protected UV induced DNA damage (10/100 μg/mL) after significant exposures. Reduced ROS generation and increased endogenous antioxidant SOD levels. Highest SPF at 1 mg/mL (18.90 ± 0.23) with good absorption in the UVA region.	[155]
	Seed	Ethanolic 75%	In vivo	Anti-oxidant and anti-inflammatory	Reduce cytokines and proinflammatory factors, and induce endogenous antioxidant protein expression	Decreased levels of NF-κB, TNF-α, IL-6 and IL-1β. Inhibition of the production of MDA and ROS. Activation of Nrf2 to induce the expression of H0-1, SOD and GSH.	[180]
	Peel	Standardized extract	In vitro and in vivo	Antioxidant, anti-hyperpigmentation and anti-tyrosinase (skin lighter)	Anti-melanogenesis and anti-tyrosinase effects	TPC: (35.91 ± 2.14) g GAE/g. DPPH: (2.29 ± 0.06) μg/mL (IC50). Tyrosinase: (197.80 ± 1.23) (IC50). No irritation observed. Litchi peel extract added to the base at 0, 0.05 or 0.1%. Skin brightening efficacy via the suppression of tyrosinase and TRP-2.	[181]
	Pericarp	Standardized extract (quercetin, rosmarinic acid and gallic acid)	In vivo	Anti-aging and anti-hyperpigmentation	Suppression of cellular melanin production via tyrosinase and tyrosinase-related protein inhibitory mechanisms; inhibition against MMP-2	Formulated into a stable non-irritating topical serum at 0.05% and 0.1%. Melanin content reduction due to its tyrosinase and TRP-2 inhibitory activities. 0.1% LC serum significantly better than control in skin lightening, skin elasticity and skin wrinkle reduction. Biological activity profile in B16F10 melanoma cells.	[154]
		Vinegar and juice	In vitro	Photoprotection	Reduce oxidative stress and proinflammatory factors and prevent photo-damage in HaCaT keratinocytes, resulting in an anti-photoaging effect	Cell viability improved after UVB exposure (vinegar 0.06% and juice 0.63%). Significant reduction of cell death after UVB irradiation. Suppression of oxidative stress by enhancing SOD and GSH-Px activity. Decrease in the mRNA level of all proinflammatory factors.	[182]
Passion Fruit	Seed	Ethanolic 96%	In vitro	Anti-acne	Antimicrobial activity against *Propionibacterium acnes*	The minimum concentration of the extract to have an antibacterial effect was 5% (MIZ: 8.5 mm). Comparable inhibitory effects with controls support its application in the management of acne vulgaris.	[159]
		40% methanolic water extract	In vitro	Photoprotection	Enhancement of the UVB protective efficacy with PFE for skin cancer and photo-damage protection	Different compositions of cosmetics with 0, 0.1 and 0.3% of PFE. DPPH (IC50): (4.41 ± 0.02) μg/mL; SPF F0.3: (9.77 ± 1.37)/M0.3: (18.99 ± 0.71)	[183]
		40% methanolic water extract divided in hexane, EtOAc and aqueous fractions	In vitro	Antioxidant and anti-tyrosinase (skin lighter)	Anti-aging and anti-wrinkle effects based on its potent antioxidant activity; UV protection efficacy and photoaging protection	TPC (EtOAc): (58.3 ± 1.1) g GAE/100 g. DPPH (EtOAc IC50): (2.7 ± 0.2) μg/mL. Antityrosinase: 39.9% (EtOAc), 33% (Aq.) SPF (EtOAc): 1.3.	[184]
		Piceatannol	In vitro	Photoprotection	Suppression of MMP-1 induction in the fibroblasts by piceatannol pre-treatment	Increased GSH level by 17% (6.25 μg/mL), 33% (12.5 μg/mL) and 77% (25 μg/mL). ROS level was decreased by 13% (0.5 μg/mL), 21% (1 μg/mL) and 58% (2 μg/mL) in irradiated keratinocytes.	[157]
Guava	Leaves	Methanolic extract	In vitro	Anti-hyperpigmentation	Anti-melanogenesis and anti-tyrosinase effects, inhibition of ORAI1 channel	Tyrosinase: 59.8% ± 1.3% (330 μg/mL). ORAI1 Inh: 80.30% ± 2.22% (100 μg/mL). ET-1 Inh: 69.96% ± 0.38%. Therapeutic potential for the treatment of melasma or the prevention of direct and indirect UV-induced melanogenesis.	[185]
		Commercial extract	In vivo	Anti-sebum	Reduction of greasiness of T-zone skin	Tannin content: (85.90 ± 1.90) mg/l. Sebum level suppression nose: 21.43 ± 3.21%; forehead: 10.72 ± 3.51% after 28 days. No irritation tested.	[164]
		Methanolic extract (triterpenes)	In vivo	Antitumor	Inhibitory effect on inflammation in ear edema and on tumor promotion	Inh. Rat. edema: 75 (1 mg/ear). *w*/*o* extract all mice had tumor, with extract 40% had tumor → 73% reduction Suppression of COX-2, TNF-α, iNOS and MMP-9.	[165]
	Pulp, peels and seeds	Water/ethyl alcohol standardized in ellagic acid	In vitro	Photoprotection	Relevant antioxidant activity	DPPH (IC50): 19.80 μg/mL. The phytocosmetic presented an increment of 17.99% of photoprotection efficacy. SPF: 22.3 ± 1.1.	[163]

Abbreviations: TPC (Total Phenolic Content), GAE (Gallic Acid Equivalent), MSE (Mango Seed Extract), ID50 (50% Infectious Dose), DPPH (Diphenyl-picrylhydrazyl), IC50 (Half maximal inhibitory concentration), MMPases (Matrix Metalloproteinases), HAase (Hyaluronidase), PFA (Polyhydric Fatty Acids), CPD (Cyclobutane Pyrimidine Dimers), TNF (Tumor Necrosis Factor), IL (Interleukin), APE (Avocado Peel Extract), ASE (Avocado Seed Extract), PPPE (Pineapple Peel Purified Extract), MIC (Minimum Inhibitory Concentration), SOD (Superoxide Dismutase), MSH (Melanocyte Stimulating Hormones), TRP (Tyrosinase Related Proteins), NRf2 (Nuclear Factor Erythroid 2 Related Factor 2), PFE (Passion Fruit Extract), SPF (Sun Protection Factor).

The cosmeceutical industry will always be in a constant search for new and improved functional ingredients from natural sources for the development of new cosmeceutical formulations. To obtain truly effective formulations, it is necessary to continue working on the optimization of the extraction conditions in order to improve the recovery of these compounds from their natural sources, observe the final effectiveness of these compounds once they are present in the cosmeceutical matrix and explore the use of innovative and advanced technologies for efficient transport, release and protection of the same. Techniques such as microencapsulation or nanotechnology are gaining special importance in the cosmeceutical industry, offering numerous advantages, such as greater bioavailability and improved release or delivery of bioactive ingredients to the skin. Therefore, future cosmeceutical products could be made by recovering and using innovative, safe and effective ingredients of natural origin incorporated in vehicles to release them in the different layers of the skin and, therefore, be able to promote regeneration and rejuvenation of skin damage produced as a consequence of environmental factors and aging effects.

## Figures and Tables

**Table 1:** Different green extraction used to obtain bioactives from tropical fruit by-products.

Fruit	By-Product	Composition Solvent	Extraction Method	Characterization Method	Major Compounds Extracted/Identified	Ref.
Mango	Seed Kernel	EtOH (50%)	Agitation for 1 h at room temperature and 2000 rpm	HPLC-UV/Vis	Gallic acid, caffeic acid, rutin, Penta-O-galloyl-b-D-glucose, galloylglucose species with galloyl moieties (greater than five)	[67]
	Peel	EtOH	UAE at 25 kHz; MAE at 2450 mHz	HPLC/ESI/MS	Phenolic compounds and flavonoids	[68]
		Lactic acid, NaOAc and H_2_O (3:1:4)	MAE at 436.45 W, 19.66 min	HPLC	Mangiferin	[69]
		EtOH	MAE + UAE (2450 MHz and 25 KHz, respectively)	HPLC/ESI/MS	Polyphenols	[70]
Pineapple	Core	Phosphate buffer	Agitation	MALDI-TOF-TOF	Glycylendopeptidases	[71]
		H_2_O	Autohydrolysis	HPLC	Gallic, hydroxybenzoic, chlorogenic, coumaric and caffeic acids and epicatechin	[72]
	Rind	N-Hexane	Cold extraction	GC-MS	Limonene, alpha-farnesene, trans caryophyllene and myrcene	[73]
Avocado	Seed	EtOH	MAE at 71.64 °C for 14.69 min	HPLC-ESI-MS	Perseitol, procyanidins, hydroxytyrosol glycoside, caffeoylquinic acid, coumaroylquinic acid, catechin and epicatechin	[74]
		EtOH/H_2_O (80:20, *v*/*v*)	UAE for 15 min at 25 °C	HPLC-MS/MS	Procyanidin B1, catechin, epicatechin and trans-5- O-cafeoyl-D-quinic acid	[26]
	Peel	EtOH (36%)	MAE for 39 min at 130 °C	HPLC-ESI-TOF/ QTOF-MS	Quinic acid, citric acid, syringic acid, procyanidins, catechin, epicatechin, rutin, quercetin derivates, naringenin and kaempferol	[75]
		MeOH (80%)	Maceration at 15 °C for 24 h	UPLC-ESI-MS/MS	Procyanidins, quinic acid, citric acid, catechin, epicatechin, rutin, quercetin, caffeic acid, ferulic acid and kaempferol	[76]
		EtOH/H_2_O (50:50, *v*/*v*)	PLE at 200 °C for 20 min at 11 MPa	HPLC-DAD-ESI-TOF-MS	Pyrocatechol, vanillic acid, catechin, epicatechin, procyanidins, rutin, quercetin, kaempferol, sakuranetina and naringenin	[77]
Custard Apple	Seed	EtOH (80%)	Agitation at 200 rpm for 1 week	GC-MS	β -stigmasterol, β -sitosterol and dihydrobrassicasterol	[41]
		MeOH/H_2_O (80:20, *v*/*v*)	UAE for 15 min at room temperature	HPLC-DAD-ESI-QTOF-MS	Catechin, epicatechin, procyanidins, vanillic acid, quinic acid, hydroxybenzoic acid and syringic acid	[78]
	Peel	MeOH (15%)	SFE with CO_2_ for 3 h at 75 °C and 100 bars	UPLC-ESI-MS	Aporphine, boldine, flaucine, anonaine, xilopine and pehnolamide	[79]
		EtOH (70%)	Agitation in the dark for 12 h at 4 °C and 120 rpm	LC-ESI-QTOF-MS/MS and HPLC-PDA	Kaphtaric acid, chlorogenic acid, p-hydroxybenzoic acid, coumaric acid, ferulic acid, sinapinic acid and resveratrol	[80]
	Leaves	EtOH/H_2_O (75:25, *v*/*v*)	Maceration for 3 days in the dark at room temperature	HPLC-ESI-DAD-MS/MS	Catequin, epicatechin, kaempferol-3- O- glucoside, anonaine, quercetin-3- O -glucoside and pronuciferin	[44]
		EtOH/H_2_O (80:20) and Acetone/H_2_O (70:30)	UAE for 20 min	HPLC-ESI-TOF-MS	Neochlorogenic acid, rutin, epicatechin, catechin, procyanidins, trigonelline, quinic acid, citric acid, kaempferol and quercetin	[81]
Papaya	Seed	CO_2_-EtOH(5, 8%)	SFE with CO_2_-EtOH for 180 min at 50 °C and 20 MPa	HPLC-ESI-MS/MS	Chlorogenic acid, caffeic acid, ferulic acid, p-hydroxybenzoic acid and p-coumaric acid	[82]
		Distilled Water	SWE for 5 min at 150 °C	LC-ESI-MS/MS	Ferulic acid, mandelic acid, vanillic acid, caffeic acid, chlorogenic acid and myricetin	[83]
	Peel	EtOH (70%)	Agitation for 12 h at 4 °C and 120 rpm	LC-ESI-QTOF-MS/MS y HPLC-PDA	Gallic acid, kaphtharic acid, *p*-hydroxybenzoic acid, syringic acid, ferulic acid, epicatechin, kaempferol and quercetin	[80]
		EtOH (23.32%)	MAE for 3 min at 340 W	GC-MS	Phenolic compounds and flavonoids	[84]
	Leaves	MeOH	Maceration for 72 h at room temperature	-	Phytol, β-sitosterol, γ-tocopherol, δ-tocopherol and hexadecanoic acid	[85]
		H_2_O	UAE for 10–20 min	HPLC-DAD-QTOF-MS/MS	Caffeoylquinic acid, coumaric acid derivatives, ferulic acid derivatives, rutin, kaempferol glucoside and rhamnoside	[86]
Guava	Seed	MeOH	Guava seed oil: Agitation for 1 min at 20 °C	RP-UHPLC-DAD-HESI-MS/MS; GC-MS	Vanillic acid, vanillin, syringaldehyde, abscisic acid and cinnamic acid, β-sitosterol, α-tocopherol, γ-tocopherol and campesterol	[87]
		EtOH/H_2_O (30:70, *v*/*v*)	UAE for 2 min at 25 °C, 20 kHz and 500 W	LC-ESI-MS/MS	Salicylic acid, cinnamic acid, p-coumaric acid, vanillic acid, ferulic acid, ellagic acid, gallic acid, galangin, naringenin, catechin and quercetin	[88]
	Peel	MeOH/H_2_O (9:1, *v*/*v*) +1% formic acid	UAE	UHPLC-DAD-MS/MS	Gallic acid and derivatives, derivatives of cinnamic acid, caffeoylquinic acids, flavanols, proanthocyanidins, ellagitannins and anthocyanidins	[89]
	Leaves	Distilled H_2_O	Boiling at 90 °C for 30 min	LC/MS	Quercetin, 3-synapoylquinic acid, esculin, gallocatequin, ellagic acid, gallic acid and citric acid	[90]
		EtOH (70%)	UAE for 30 min at 340 W	HPLC-ESI-TOF-MS	Rutin, quercetin, quercitrin, kaempferol and avicularin	[91]
Litchi	Seed	EtOH (95%)	Direct reflux extraction for 2 h at 90 °C	HPLC	Catechin, epicatechin, litchiol A and litchiol B	[92]
	Peel	Phosphate buffer Hot H_2_O	Enzyme extractionPressurized hot-water extraction	HPLC	Polyphenolic content	[93]
Pass. Fruit	Seed	EtOH (70%)	Agitation for 30 min at 80 °C	HPLC-DAD LC-ESI-MS/MS	Flavonoids	[94]
	Peel	EtOH (70%)	HAE for 2 min	UHPLC-PDA	Isoorientin, orientin and sovitexin	[15]
	Leaves	Aqueous solutions of ionic liquids (ILs)	IL-MA-SLE	HPLC-PDA	Rutin, quercetin and apigenin	[95]

Abbreviations: EtOH (Ethanol), HPLC (High Performance Liquid Chromatography), Vis (Visible), UAE (Ultrasound-Assisted Extraction), MAE (Microwave-Assisted Extraction), ESI (Electrospray Ionization), MS (Mass Spectrum), NaOAc (Sodium Acetate), MALDI (Matrix-Assisted Laser Desorption/Ionization), TOF (Time Of Flight), GC (Gas Chromatography), QTOF (Quadrupole Time Of Flight), MeOH (Methanol), UPLC (Ultra Performance Liquid Chromatography), PLE (Pressurized Liquid Extraction), DAD (Diode-Array Detection), SFE (Supercritical Fluid Extraction), LC (Liquid Chromatography), PDA (Photodiode-Array Detection), SWE (Subcritical Water Extraction), RP (Reversed Phase), UHPLC (Ultra High Performance Liquid Chromatography), HESI (Heated Electrospray Ionization), HAE (Homogenizer-Assisted), IL-MA-SLE (Ionic Liquid Microwave-Assisted Solid-Liquid Extraction).
